# Immunological and polyfunctional T cell profiling following therapy with DPX-Survivac as a predictive tool of vaccine efficacy

**DOI:** 10.1186/2051-1426-1-S1-P106

**Published:** 2013-11-07

**Authors:** Mohan Karkada, Mieko Ogura, Vivian Tovar, Masato Mitsuhashi, Neil Berinstein, Marc Mansour

**Affiliations:** 1 Immunovaccine Inc., Halifax, NS, Canada; 2 Hitachi Chemical Research Center Inc., Irvine, CA, USA; 3 Sunnybrook Health Sciences Centre, Toronto, ON, Canada

## 

Predicting the likelihood of a clinical outcome using biomarkers during the course of a cancer immunotherapy can be a powerful tool for time-sensitive decision making on personalized treatment options. In a clinical trial with the survivin-targeting vaccine DPX-Survivac, we showed using established methods (ELISpot/tetramer/flow cytometry) that a high-dose of DPX-Survivac vaccine in combination with low dose metronomic oral cyclophosphamide (mCPA) induced much stronger immune responses in ovarian cancer patients (cohort C, n=6) compared to vaccine alone (cohort A, n=6) or low dose vaccine with mCPA (cohort B, n=6). Immune responses were detected ex vivo in patients’ PBMCs particularly in cohort C, a reflection of the magnitude of the immune responses generated by the vaccine. We further analyzed the kinetics of various T cell phenotypes of the CD4 and CD8 lineages, including effector/ central memory T cells and late differentiated CD8 T cells. We established a model for the sequential appearance of polyfunctional T cells of the various phenotypes and for the persistence of these cell types following repeated immunizations with DPX-Survivac. In addition, we explored a unique PCR-based ‘real-time’ method developed by Hitachi Chemical Research Center for analyzing mRNA expression signatures that could be associated with vaccine induced immune responses. For this, small quantities of fresh blood from immunized patients were directly incubated with vaccine antigens and analyzed by qPCR at pre- and one month post-immunization time points. Among 9 immune response-related biomarkers tested, mRNA for molecules such as IFN-γ, GM-CSF, TNF-α and CXCL10 were detected de novo in the blood of four out of four subjects tested among six patients from cohort C at one month following the third vaccination. These results are in keeping with the establishment of stronger immune responses and the kinetics of vaccine-induced memory/effector T cells observed at this time point in patients receiving the combination of DPX-Survivac and cyclophosphamide. Taken together, these findings provide a framework for identifying a potential immune response signature soon after initiation of the vaccine therapy. We will determine whether this immune response signature is predictive of clinical activity in a planned randomized phase 2 trial.

